# Asymmetric stem-loop–mediated isothermal amplification of nucleic acids for DNA diagnostic assays by simple modification of canonical PCR primers

**DOI:** 10.3389/fbioe.2022.931770

**Published:** 2022-07-22

**Authors:** Rui Mao, Xinyao Wu, Qing Miao, Ting Cai

**Affiliations:** ^1^ Key Laboratory of Diagnosis and Treatment of Digestive System Tumors of Zhejiang Province, Hwa Mei Hospital, University of Chinese Academy of Sciences, Ningbo, China; ^2^ Ningbo Institute of Life and Health Industry, University of Chinese Academy of Sciences, Ningbo, China

**Keywords:** nucleic acid detection, isothermal amplification, asymmetric stem-loop, point of care test, real-time fluorescence, endpoint colorimetric

## Abstract

Nucleic acid–based assays have been adopted as mainstream tools for clinical diagnostics, food safety, and environment monitoring with the merits of accuracy, rapidity, and sensitivity. Loop-mediated isothermal amplification (LAMP) is a well-established method to rapidly identify nucleic acids and has gained recognition and been developed for clinical applications in resource-limited areas. However, the needs for specifically designed primer sets and non-specific amplification hinder the development of LAMP-based nucleic acid tests. Here, a promoted method, termed asymmetric stem-loop–mediated isothermal amplification (ASLAMP) by simple modification of canonical PCR primers, was developed to attempt to overcome those drawbacks. The two primers in the ASLAMP reaction can be easily obtained by adding a stem-loop sequence part to one PCR primer at 5′-ends to get the folding primer (FP), then adding the same primer to the counter canonical PCR primer at 5′-ends to get the turn-back primer (TP). The ASLAMP method was demonstrated in detecting the H1N1 gene fragment with merits of simple primer design, short target sequence, and high amplification efficiency. In addition, the ASLAMP method showed similar efficacy compared with LAMP targeting at the same H1N1 gene sequence. Furthermore, *Shigella* detection monitored by real-time fluorescence and endpoint colorimetric approaches were taken as examples for evaluation of the practical application of the ASLAMP method, both offered 100% sensitivity and specificity. In conclusion, the novel ASLAMP method with simplicity of primer design, low requirement of equipment, efficiency, and rapidity has exhibited its great prospect for establishment of DNA isothermal amplification in point of care application.

## Introduction

Nucleic acid–based assays have been adopted as mainstream tools for clinical diagnostics, food safety, and environment monitoring with the merits of accuracy, rapidity, and sensitivity. Among those approaches, polymerase chain reaction (PCR), the most practical, rapid, accessible, and accurate molecular diagnostic approach, has been developed for identification of interested targets (Saiki et al., 1985; Petralia and Conoci, 2017). In particular, PCR-based detection methods have facilitated appropriate responses to the emerging viral threats, such as, avian influenza A viruses, Ebola virus, and most recently SARS-CoV-2 pandemic (Gao, 2018; Lu et al., 2020; Valera et al., 2021). With the merits of low hardware dependence and rapid amplification of nucleic acid sequences at constant temperature, lots of isothermal amplification techniques have been invented to supplement the sophisticated PCR method (Ju et al., 2021). Recently, novel methods, such as, cross-priming amplification (CPA) (Fang et al., 2009), polymerase spiral reaction (PSR) (Liu et al., 2015), competitive annealing–mediated isothermal amplification (CAMP) (Mao et al., 2018), and closed dumbbell–mediated isothermal amplification (CDA) (Mao et al., 2022), have been extensively established and tried to apply into clinical practice. Among those well-established isothermal amplification methods, LAMP, with the advantages of robustness, rapidity, specificity, and sensitivity, has been verified in many systems for pathogen diagnosis (Zhang Y. et al., 2014; Landaverde et al., 2020; Sheikh et al., 2020; Wang T. et al., 2021; Koeck et al., 2021). However, the drawbacks of specifically designed four to six primers and non-specific amplification hindered the development of LAMP-based nucleic acid tests. (Kimura et al., 2011; Chang et al., 2013; Su et al., 2015; Newbigging et al., 2019; Nguyen et al., 2020).

**FIGURE 1:**
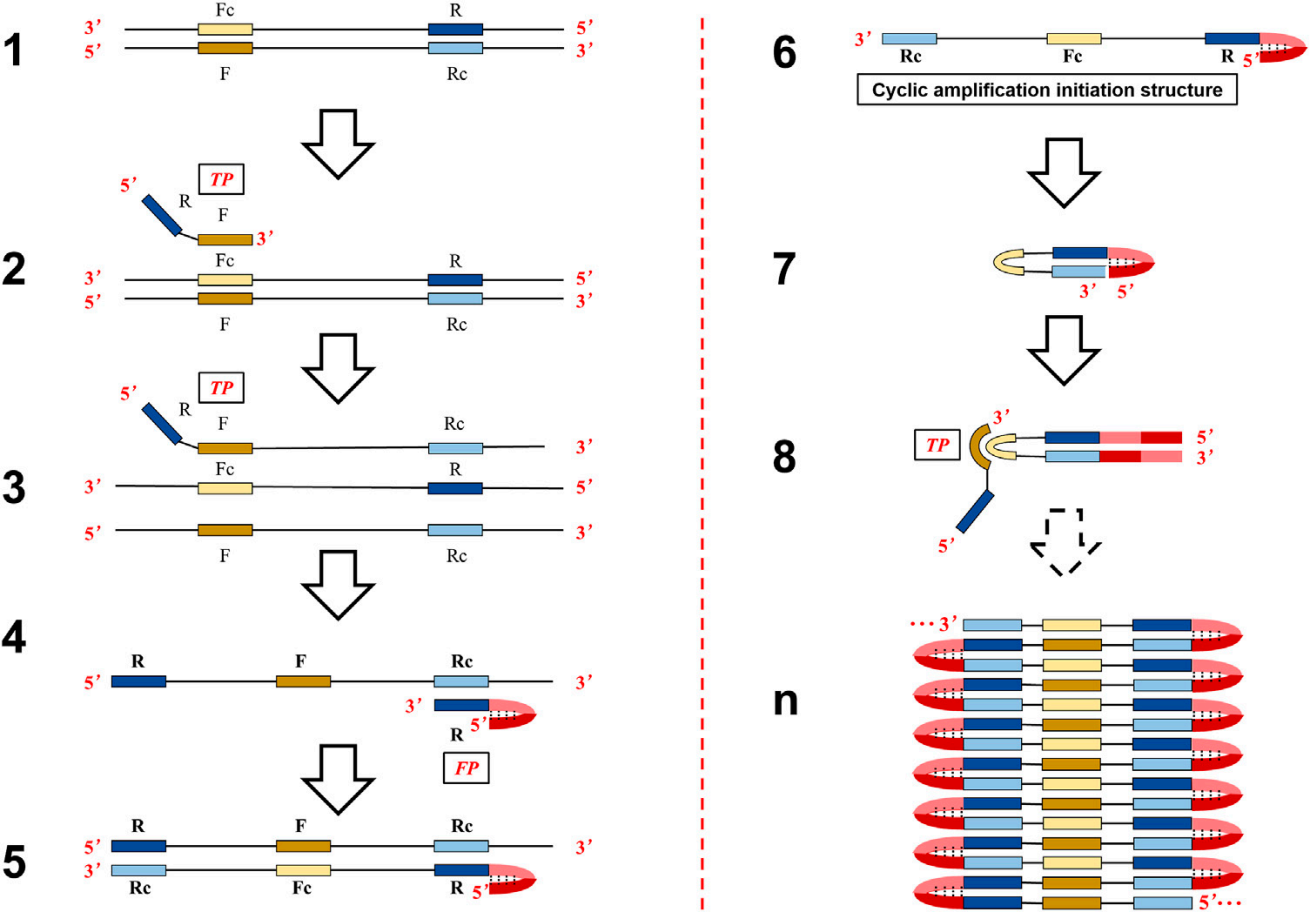
Schematic presentation of the mechanism of the ASLAMP method.

The objective of this study was to offer an option to solve problems of difficult and cumbersome primer set design of LAMP and try to decrease non-specific amplification. In particular, similar isothermal amplification based on self-primed nucleic acid structure (asymmetric stem-loop or dumbbell structure–based isothermal amplification of nucleic acids, ASLAMP) was established by simple modification of canonical PCR primer. The theoretical reaction steps of ASLAMP are represented in Figure 1. When the reaction is conducted at 60–65°C, the double-stranded structure of template DNA is unstable and could unlock automatically in the presence of betaine (Fei and Ha, 2013). The F segment of the TP primer anneals to Fc in single-stranded–targeted DNA and starts strand extension (structure 2 to structure 3). The Rc sequence in the newly synthesized TP-linked complementary strand is annealed by R in FP to extend (structure 4 to structure 5), then a single chain is obtained and released (structure 6). The single-stranded nucleic acid in structure 6 (3′-Rc-Fc-R-Dc-D-5′) can form an asymmetric dumbbell-like structure, that is, a hairpin structure at the 5′ end of the D-Dc sequence and the Rc at the 3′ end can reversely complementary to the inner R region (structure 7). The structure 7 is similar to the dumbbell-like structure which serves as a starting template to initiate self-primed DNA synthesis in LAMP (Tomita et al., 2008). Thus, the 3′ end can start DNA synthesis by the strand displacement process when the Rc region of 3′ end is annealed to the R region. At the same time, the F in the TP primer is hybridized to single-stranded Fc sequence in the loop to extend (structure 8). The cycling amplification steps of ASLAMP using self-structure as the templates to achieve nucleic acid synthesis were similar to LAMP (Supplementary Figures S1C,D depict the similarity of start structure for self-extension between LAMP and ASLAMP). Finally, similar to the self-extension and primer-aided nucleic acid synthesis procedures in LAMP, a mixture of amplification products featured by adjacent annealed DNAs are obtained (structure n) by ASLAMP. Given the mechanism described earlier, the length of the targeted DNA region required for primer design of ASLAMP can be as short as 40 bps.

The principle, performance, and non-specific reaction of the newly ASLAMP method have been investigated by amplifying the targeted H1N1 gene fragment. Also, the boost primer was designed and selected for the H1N1 gene to get a boost-ASLAMP (B-ASLAMP) reaction, which would dramatically reduce the reaction time by accelerating DNA synthesis without non-specific amplification. In addition, the amplification efficiency of ASLAMP was comparable with LAMP in detecting the same H1N1 target. Moreover, the visible onsite ASLAMP detection approach by hydroxy naphthol blue (HNB) endpoint indication was also verified. Furthermore, real-time fluorescence and HNB-based endpoint colorimetric ASLAMP detection of *Shigella* were taken as instances to obtain practical application assessment with both 100% sensitivity and specificity. In conclusion, all results suggested great potential of the developed ASLAMP method to achieve a simple, easy-to-operate, and fast nucleic acid analysis tool for the point of care diagnosis.

## Materials and methods

### Reagents and materials

DNA plasmids and primers were provided by BGI Biological Engineering Technology and Services Co., Ltd. (Shenzhen, China). DNA fragments of the H1N1 gene (GenBank: GQ290690.1) and *Shigella* gene (GenBank: CP055125.1) were cloned into pMV vectors to construct plasmid DNA templates. The *Bst* 2.0 WarmStart DNA polymerase and 10× ThermoPol reaction buffer (including 200 mM Tris-HCl, 100 mM KCl, 100 mM (NH_4_)_2_SO_4_, 20 mM MgSO_4_, and 1% Triton X-100) were purchased from New England BioLabs (Ipswich, MA, United States). The DNA molecular weight marker was purchased from Thermo Fisher Scientific (Waltham, MA, United States). Eva Green and GelRed were obtained from Biotium (Hayward, CA, United States). Deoxynucleotide triphosphates (dATP, dTTP, dGTP, and dCTP), restriction enzyme *EcoRV*, DNA purification kits, and DNA extraction kits were purchased from Sangon Biotech (Sangon, Shanghai, China). Other reagents, unless specified, were obtained from Sigma-Aldrich (St. Louis, MO, United States). The online DNA copy number calculator (http://cels.uri.edu/gsc/cndna.html.) was adopted to determine copy numbers of targets.

### ASLAMP and B-ASLAMP reaction

The ASLAMP assays were conducted in 25 μl reaction mixtures with the following components: 8 U *Bst* 2.0 WarmStart DNA polymerase, 2.5 μl 10 × ThermoPol reaction buffer, 1 M betaine, 6 mM MgSO_4_, 1.4 mM of each dNTP, 1.6 μM of TP and FP, and an appropriate amount of the nucleic acid sample. For the B-ASLAMP reaction, another BP (0.8 μM) was added to the mixtures. Negative control (NC) contained non-target nucleic acid samples or nuclease-free water. The reactions were generally carried out at 63°C for 60 min and heating at 85°C for 10 min to terminate.

Similar to LAMP, the by-products of insoluble magnesium pyrophosphate were produced in the process of ASLAMP amplification, and hydroxy naphthol blue (HNB, final concentration 120 μM) was added to indicate Mg^2+^ reduction in the reaction mixtures described earlier to achieve endpoint monitoring. Positive ASLAMP and B-ASLAMP colorimetric reactions are indicated by endpoint blue, while negative reactions keep violet in HNB-added reaction mixtures.

### LAMP reaction

The LAMP reaction was conducted using the identical mixture applied in ASLAMP except for the primers. The LAMP and ASLAMP were performed with closely matched primer sets targeting the H1N1 gene. The primer set for LAMP consists of forward inner primer (FIP), backward inner primer (BIP), forward outer primer (F3), and forward outer primer (B3). The primers needed for the H1N1 LAMP reaction are 1.6 μM for FIP and BIP and 0.2 μM for F3 and B3 (Supplementary Table S1). For the amplification efficiency comparison, LAMP, ASLAMP, and B-ASLAMP reactions were prepared at the same time and performed at 63°C for 60 min in a Real-Time PCR detection system using identical 10^4^ copies of the H1N1 plasmid gene.

### Amplification product analysis

The ASLAMP reaction was real-time monitored by Eva Green which would emit fluorescence when intercalating with dsDNA. The instrument SLAN-96 Real-Time PCR detection system (Sansure biotechnology, Changsha, China) was set to measure the fluorescence intensity in every minute intervals. After ASLAMP and B-ASLAMP reaction, 5 μl products were electrophoresed at 70 V in 1% agarose gel, stained with GelRed and visualized by the ChemiDoc™ XRS Imaging System.

### Restriction enzyme analysis of H1N1 gene ASLAMP products

**FIGURE 2:**
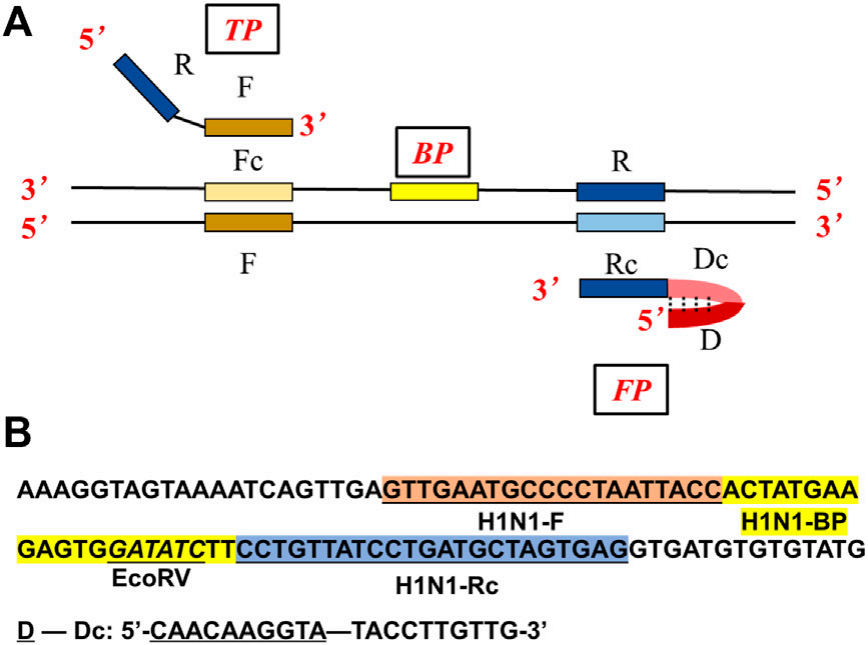
**(A)** Primer design of the ASLAMP method. **(B)** Localization of primers and restriction enzyme cutting site in the nucleotide sequence of the modified H1N1 gene.

The products of ASLAMP and B-ASLAMP methods targeting the modified H1N1 gene fragment which has a restriction enzyme recognition site for *EcoRV* (Figure 2B) were purified using commercial kits. Then, the purified products were digested with enzyme *EcoRV* at 37°C following the instruction. Then, the digested and pro-digested products by H1N1 gene ASLAMP and B-ASLAMP amplification were analyzed using 1% agarose gel electrophoresis.

### Real-time and colorimetric detection of *Shigella* by ASLAMP

The ASLAMP-based *Shigella* assays by real-time fluorescence and endpoint colorimetric approaches were taken as instances of practicability investigation. For pathogen DNA preparation, single clones of the bacteria were selected from LB agar plates and cultured in 5 ml LB broth at 37°C overnight by a shaker incubator (180 rpm). For further exploration of the practical application, single clones of the standard strain of *Shigella* were mixed with pasteurized milk (5 ml) and smashed bread (5 g) in sterilized 50 ml corning tubes at room temperature overnight to mimic the natural process of infection. After incubation, the liquid cultures and artificial infections were collected and extracted using commercial DNA extraction kits or easily by boiling. The sensitivity of the developed *Shigella* ASLAMP method was evaluated using 10-fold serial dilutions of the strain ranging from 1.0 × 10^6^ to 10 copies/μl. Also, the specificity of the *Shigella* B-ASLAMP assay was further explored using DNA samples extracted from cultured standard strains of *Escherichia coli* (CVCC 1491), *Salmonella* (CVCC1789), *L. monocytogenes* (CVCC 1597), and *Vibrio parahaemolyticus* (CGMCC 1.1997) strains (CVCC: China Veterinary Culture Collection Center, CGMCC: China General Microbiological Culture Collection Center).

## Results

### The principle of the ASLAMP method

Similar to LAMP, ASLAMP is dependent on auto-cycling DNA synthesis by strand displacement activity of DNA polymerase, but just needs a pair of specially designed primers (TP: turn-back primer, FP: folding primer in Figure 2A). Compared to conventional LAMP primer design (Supplementary Figure S1A), the primers can be easily designed from canonical PCR primers (typically 18–22 nts) of the targeted sequences. As depicted in Figure 2A, the sequences of F and R stands for the “forward” and “reverse” primer sequences in PCR respectively, the sites with lowercase “c” represent “complementary” sites. Based on this structure, the primers of TP and FP are designed as follows. TP contains the sequence R and the sequence F. FP contains the external sequence D-Dc (D stands for non-sense) and the sequence R at the 3′ end. Boost primer is situated between F and Rc. The F sequence, R sequence, and boost sequence should be 17–24 base pairs with a GC rate of 45%∼55% like the requirement of PCR reaction. We can see that the D-Dc is self-annealing and could form a hairpin structure at the 5′ end. Notably, the external sequence D-Dc is optional at any times which would offer an option to adjust theoretically and practically. So, the interaction of primers which caused non-specific amplification can be eliminated (Rolando et al., 2020).

### H1N1 gene amplification by ASLAMP

**FIGURE 3:**
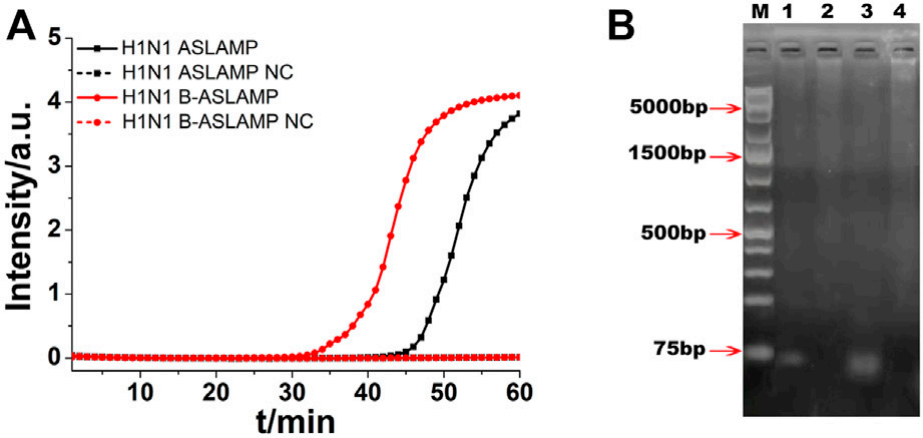
**(A)** ASLAMP and B-ASLAMP amplifications monitored by real-time PCR carried out at 63°C for 60 min. **(B)** Gel electrophoresis results for ASLAMP and B-ASLAMP products prior and after restriction enzyme digestion. Line M, molecular weight marker. Lane 1, H1N1 ASLAMP product that has been digested by *EcoRV*. Lane 2, ASLAMP products prior to digestion. Lane 3, B-ASLAMP product that has been digested by *EcoRV*. Lane 4, B-ASLAMP products prior to digestion. NC is the abbreviation of negative control.

To demonstrate the principle of ASLAMP, the H1N1 gene fragment modified by adding a restriction site (*EcoRV*) sequence was adopted as a model for illustration. The ASLAMP primers of the H1N1 gene are exhibited in Figure 2B and Supplementary Table S1 based on canonical H1N1 PCR primer pairs accordingly. The reactions were conducted by addition of a primer mixture of 40 μM TP and FP at constant 63°C for 60 min. The real-time fluorescence curve showed that two primers of ASLAMP successfully amplified 100 copies of the H1N1 gene fragment (Figure 3A).

After real-time fluorescence analysis, the electrophoresis of H1N1 ASLAMP products by 1% agarose gel was operated. The results showed that the amplification products were ladder-like bands with various molecular weights (lanes 1 and 2 in Figure 3B). The minimum band was consistent with the theoretical monomeric amplicons primed by TP and FP. Also, other larger bands were probably multiple-unit amplicons matched with the expected ASLAMP products. The gel electrophoresis demonstrated the correctness of the ASLAMP principle.

### Optimization of H1N1 gene ASLAMP reaction

**FIGURE 4:**
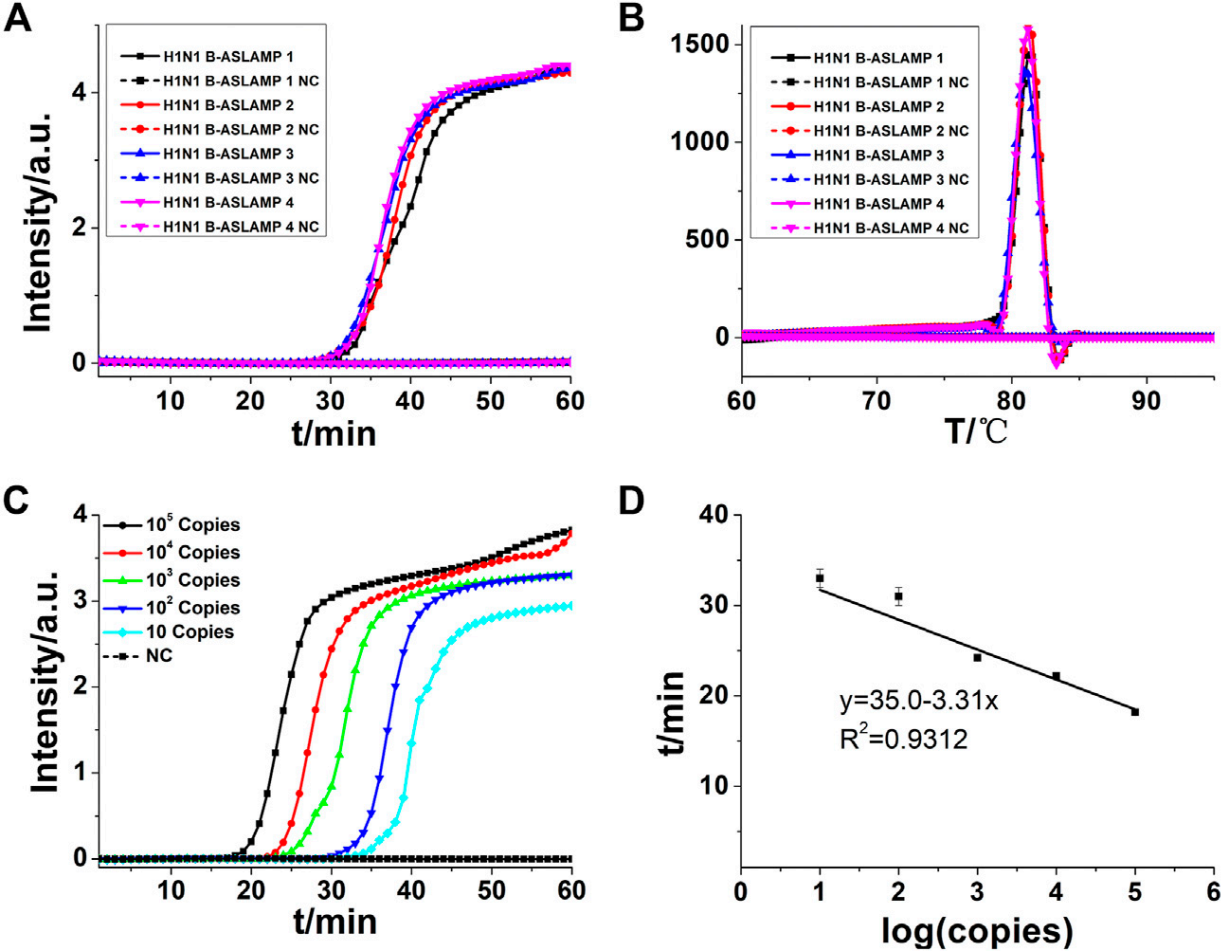
**(A)** B-ASLAMP amplification monitored by real-time PCR. **(B)** Melting curve analysis of B-ASLAMP products by real-time PCR. **(C)** H1N1 gene amplifications of serial dilutions of the H1N1 gene by B-ASLAMP monitored by real-time PCR carried out at 63°C for 60 min. **(D)** Standard curve generated by B-ASLAMP amplification of serial dilutions of the H1N1 gene. NC is the abbreviation of negative control.

The H1N1 DNA gene was also used for the illustration of boost primer with ASLAMP (Figure 2A exhibits the location of turn-back primer, folding primer, and boost primer; Figure 2B shows the primer location targeting at H1N1 gene), termed as B-ASLAMP. The threshold time was shortened dramatically in detection of 100 copies of the H1N1 gene by B-ASLAMP compared with ASLAMP (Figure 3A), which indicated dramatic improvements in amplification efficiency. Moreover, the boost primer exhibited improvement of efficiency in amplification of low concentration of target DNA (Figure 4C). Meanwhile, the threshold detection time by H1N1 gene B-ASLAMP amplification for 100 copies was 29.5 min, and the melting curve analysis for the products was 81.25°C as shown in Figures 4A,B. In addition, both the real-time fluorescence analysis and melting curve analysis of the H1N1 DNA gene amplification displayed the repeatability of the B-ASLAMP method (4 repeats).

To detect limitation, the cycle threshold (Ct) value of 10 copies of the H1N1 gene amplified by B-ASLAMP was about 33 min as shown in Figure 4C, demonstrating the fine sensitivity and efficiency of the novel method. In addition, the standard curve depicted by plotting the value of Ct vs. copy number (Figure 4D) indicated that the H1N1 B-ASLAMP method held a potential for semi-quantification of target DNA (*R* ^2^ = 0.9312).

### Restriction enzyme analysis of ASLAMP and B-ASLAMP products

The modified H1N1 gene fragment with a restriction enzyme recognition site of *EcoRV* was used for demonstration of ASLAMP and B-ASLAMP method, and the properties of the developed methods were further explored by restriction enzyme analysis of the amplification products. The result showed that after digestion, the ASLAMP and B-ASLAMP products of various molecular weights were split into single short DNA and the theoretical digestion products were 74 bp pieces (Figure 3B, lanes 2 and 4). The specificity and principle of the boost ASLAMP (B-ASLAMP) reaction were also demonstrated by the electrophoresis and threshold time of the real-time curve of the both methods. Notably, false positive results were not observed with the addition of boost primer. Therefore, the extra addition of boost primer in the ASLAMP reaction was helpful to realize high specificity and efficiency for amplification of DNA.

### Comparison of ASLAMP and LAMP in amplification of the H1N1 gene

**FIGURE 5:**
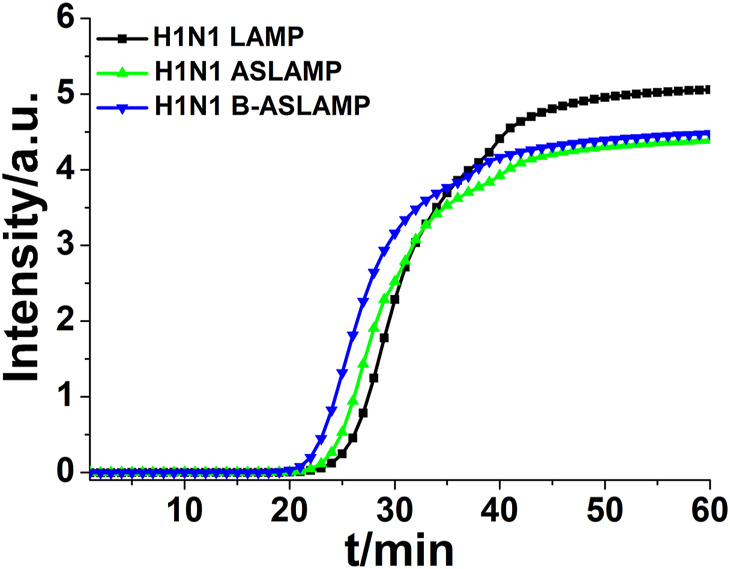
Real-time fluorescence curve of LAMP, ASLAMP, and B-ASLAMP amplification of the H1N1 gene.

From the amplification principle, the newly established ASLAMP method could be classified as a promoted LAMP method. Loop structures in both methods were of great importance to innate rapid accumulation of target nucleic acid signals. To obtain a primary evaluation of amplification efficiency of the developed ASLAMP assay, the same concentration of the H1N1 gene fragment was adopted to get amplified by the LAMP, ASLAMP, and B-ASLAMP assays (the sequences of primers are listed in Supplementary Table S1). The B-ASLAMP primer sets successfully amplified same 10^4^ copies of the target with the lowest threshold time (Ct) compared with LAMP and the ASLAMP (Figure 5), indicating highest efficiency in H1N1 gene detection.

### Colorimetric endpoint monitor approach of the ASLAMP method

**FIGURE 6:**
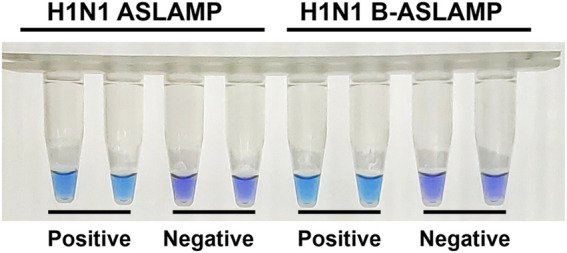
HNB-based colorimetric ASLAMP, and B-ASLAMP assays for H1N1 gene detection.

Visual detection tools allowed us to judge the existence of interested biomarkers in low-infrastructure areas where there was lack of bulky and expensive analytical instrumentations. Various visually monitoring innovations have been developed for the well-established LAMP (Zhang X. Z. et al., 2014; Fischbach et al., 2015). To facilitate onsite ASLAMP detection similarly, the colorimetric detection method was explored. In this study, an identical endpoint visual monitoring by HNB indicated that the Mg^2+^ reduction approach was adopted for the ASLAMP reaction. As shown in Figure 6, the HNB-based H1N1 ASLAMP and B-ASLAMP colorimetric reactions conducted at 63°C for 60 min can be judged by naked eyes. Positive ASLAMP and B-ASLAMP colorimetric reactions are indicated by endpoint blue, while negative reactions keep violet in HNB-added reaction mixtures.

### ASLAMP for *Shigella* detection

**FIGURE 7:**
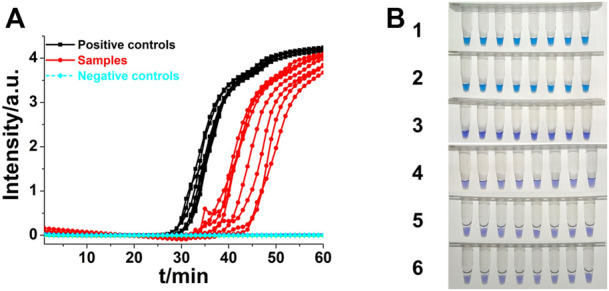
**(A)** Real-time B-ASLAMP for *Shigella* positive controls, samples and negative control (*E. coli*, *L. monocytogenes*, *Salmonella*, and *V. parahaemolyticus*). **(B)** Colorimetric B-ASLAMP based on HNB. Line 1, positive controls (10^5^ copies of templates). Line 2, samples extracted from the standard strain of *Shigella*. Line 3, samples extracted from the standard strain of *E. coli*. Line 4, samples extracted from the standard strain of *L. monocytogenes*. Line 5, samples extracted from the standard strain of *Salmonella*. Line 6, samples extracted from the standard strain of *V. parahaemolyticus*.

*Shigella* is an important foodborne pathogen that usually causes clinically severe diarrhea (Zhang et al., 2018). Real-time fluorescence and HNB-based endpoint colorimetric ASLAMP detection of *Shigella* were taken as instances to further exploration of this newly developed approach. The *Shigella* ASLAMP assays were conducted using high qualified DNA samples extracted by commercial kits and crude DNA samples prepared by simple boiling and quick centrifugation (the sequences of primers are listed in Supplementary Table S2). Real-time fluorescence curves showed that the developed *Shigella* B-ASLAMP system successfully amplified the target gene sequence of extracted genome samples including artificially contaminated samples (Figure 7A). No cross-reaction was observed when using the *Shigella* B-ASLAMP system to amplify genome DNA samples extracted by commercial kits from cultured strains of *E. coli*, *Salmonella*, *Listeria monocytogenes*, and *V. parahaemolyticus*. For endpoint colorimetric judgment by HNB-based *Shigella* B-ASLAMP assay (Figure 7B), all samples were determined correctly (100% success). The detection limitation of B-ASLAMP *Shigella* assay was 10 copies within 50 min (Supplementary Figure S2), showing good amplification efficiency. Furthermore, *Shigella* detection monitored by real-time fluorescence and endpoint colorimetric approaches both offered 100% sensitivity and specificity. (Table 1). All results proved that the developed B-ASLAMP method could detect target DNA of *Shigella* robustly and rapidly.

**TABLE 1:** Sensitivity and specificity of B-ASLAMP assay for *Shigella*.

Species	Sample number		Sensitivity	Specificity	Accuracy
	*Shigella* B-ASLAMP	Defined	95% CI^a^	95% CI^a^	95% CI^a^
*Shigella*	80	80	1.0 (95.5–100.0)	1.0 (97.2–100.0)	1.0 (98.2–100.0)
*E. coli*	0	32			
*L. monocytogenes*	0	32			
*Salmonella*	0	32			
*V. parahaemolyticus*	0	32			
Total	80	208			

a CI, confidence interval. Statistical analysis was carried out by the online program of “Diagnostic test evaluation calculator,” https://www.medcalc.org/calc/diagnostic_test.php.

## Discussion

Nucleic acid amplification–based techniques played critical roles in developing rapid and accurate on-site diagnosis of infectious diseases (Lee et al., 2021). Identification of specific genes of pathogens by probe-based real-time fluorescence PCR accelerated the control of the culprit SARS-CoV-2 pandemic (Valera et al., 2021). Various LAMP-based approaches were developed to achieve the point of care test of SARS-CoV-2 (Dao Thi et al., 2020; Wang R. et al., 2021; Zhang et al., 2021). However, the drawbacks of cumbersome primer design and false-positive amplification hindered the development of LAMP-based nucleic acid tests.

In this research, a novel isothermal amplification–based on self-primed nucleic acid structure (asymmetric stem-loop or dumbbell structure–based isothermal amplification of nucleic acids, ASLAMP) was established by simple modification of canonical PCR primer. This approach would offer an alternative method of LAMP for rapid isothermal detection of nucleic acids by simple modification of canonical PCR primer. As the primer design and amplification principle demonstrated, ASLAMP should be termed as a variation of the LAMP method. The principle of ASLAMP was demonstrated by amplification of the modified H1N1 gene fragment with merits of simple and high efficiency. The study of reproducibility of B-ASLAMP was illustrated by both real-time fluorescence curve and melting curve analysis. In addition, the plot of the value of threshold time (Ct) versus copy number of the H1N1 gene plasmid indicated the developed B-ASLAMP would be applied for semi-quantification of target DNA. The results of colorimetric assays were in accordance with real-time fluorescence assays in amplification of the H1N1 gene, indicating on-site detection potential of the HNB-based ASLAMP method.

After all, the ASLAMP method showed similar amplification efficacy compared with LAMP. The ASLAMP needed a simpler primer design procedure and shorter target sequence compared with LAMP as shown in Supplementary Figures S1A,B. In addition, a similar starting structure for self-extension of LAMP and ASLAMP indicated the developed assays would obtain similar amplification efficiency in theory (Supplementary Figures S1C,D). In particular, a simple and easy primer design process of ASLAMP would achieve a convenient establishment of an isothermal amplification system from the existing PCR reaction system. Furthermore, HNB-based colorimetric B-ASLAMP would help to facilitate the development of onsite *Shigella* detection which exhibited 100% sensitivity and specificity.

In conclusion, with the advantages of simple primer design and no need of outer primer, ASLAMP has the potential to realize simple and fast nucleic acid amplification–based analysis (Liu et al., 2018; Yamanaka et al., 2018; Varona and Anderson, 2019). Single nucleotide polymorphism (SNP) identification and other applications by ASLAMP should be explored in near future (Mitani et al., 2007). It is hopeful that the established ASLAMP technique could develop into a powerful point of care DNA assays for rapid, simple, reliable, and sensitive diagnosis.

## Data Availability

The original contributions presented in the study are included in the article/Supplementary Material; further inquiries can be directed to the corresponding authors.
