# Supplementary material for: Asymmetric stem-loop–mediated isothermal amplification of nucleic acids for DNA diagnostic assays by simple modification of canonical PCR primers
Source: Front Bioeng Biotechnol. 2022 Jul 22;10:931770. doi: 10.3389/fbioe.2022.931770 (PMC9355699; doi:10.3389/fbioe.2022.931770)
Supplement: Supplementary file 1 [file DataSheet1.docx]

Supplementary Material

**Asymmetric stem-loop mediated isothermal amplification of nucleic acids for DNA diagnostic assays by simple modification of canonical PCR primers**

Rui Mao^a, b^*, Xinyao Wu^a, b^, Qing Miao^a, b^ and Ting Cai^a^^, b^*

^a^ Key Laboratory of Diagnosis and Treatment of Digestive System Tumors of Zhejiang Province, Department of Experimental Medical Science, Hwa Mei Hospital, University of Chinese Academy of Sciences, Ningbo 315010, China.

^b^ Ningbo Institute of Life and Health Industry, University of Chinese Academy of Sciences, Ningbo 315010, China.

*To whom correspondence should be addressed:

E-mail: [maorui@ucas.ac.cn](mailto:maorui@ucas.ac.cn), [caiting@ucas.ac.cn](mailto:caiting@ucas.ac.cn)

Phone: (86) 0574-83870266

Full address: No. 41 Xibei Street, Haishu District, Ningbo, Zhejiang, 315010, P.R. China.

**Supplementary Table S1**. Primer sequences of ASLAMP and LAMP targeting the H1N1 gene (GenBank: GQ290690.1)

| Method | Primer | Sequence (5´→3´) |
| --- | --- | --- |
| ASLAMP | H1N1-TP | CTCACTAGCATCAGGATAACAGG-GTTGAATGCCCCTAATTACC |
|  | H1N1-FP | CAACAAGGTATCCTTGTTG-CTCACTAGCATCAGGATAACAGG |
|  | H1N1-BP | ACTATGAAGAGTGGATATCTT |
| LAMP | H1N1-FIP | TAAAGCAAGAACCATTAATGCAGGC-TTGGAGAAACAACATATTGAGG |
|  | H1N1-BIP | CCATAATGACTGATGGACCAAGT-AACTGATTTTACTACCTTTCCCTT |
|  | H1N1-F3 | GGCATAATAACAGATACTATCAAGA |
|  | H1N1-B3 | AGGAACACTCTTCATAGTGG |

**Supplementary Table S2**. Primer sequences of ASLAMP targeting the *Shigella* gene (GenBank: CP055125.1)

| Method | Primer | Sequence (5´→3´) |
| --- | --- | --- |
| ASLAMP | Shigella-TP | GGAAAAACTCAGTGCCTC-TCTCAGTGGCATCAGCAG |
|  | Shigella-FP | CAACAAGGTATCCTTGTTG-GGAAAAACTCAGTGCCTC |
|  | Shigella-BP | TTCGACAGCAGTCTTTCG |


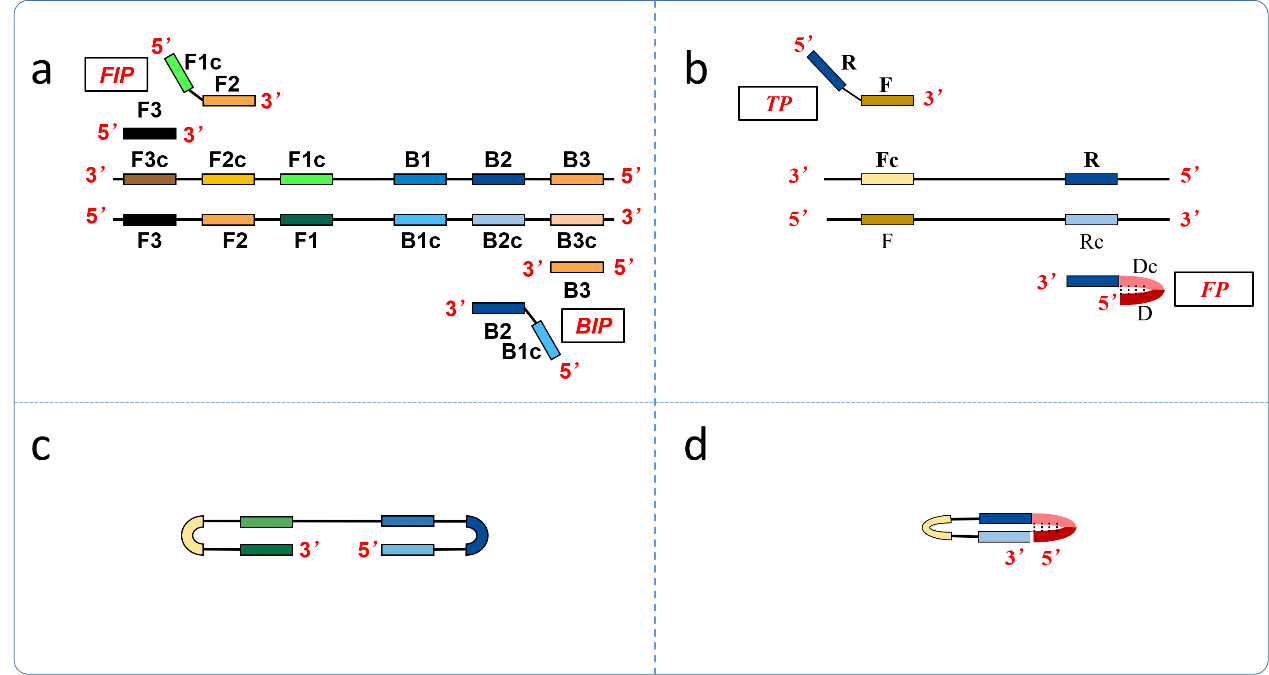

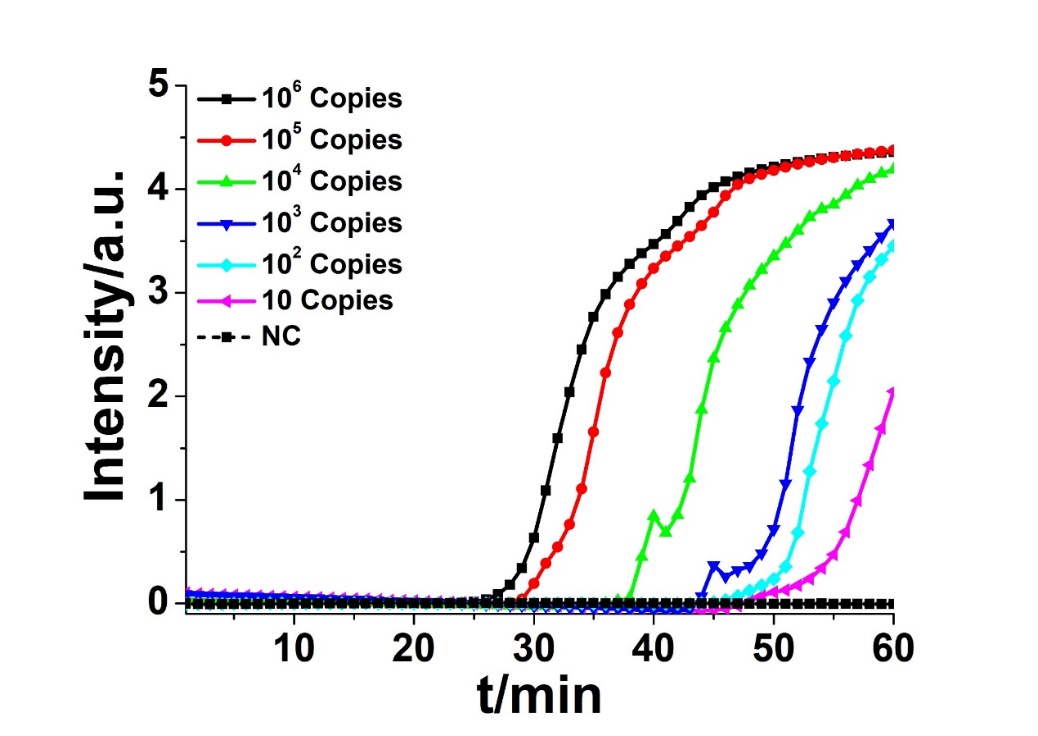


**Supplementary Figure S1** Comparison of LAMP and ASLAMP method. (a) Primer design and locations of LAMP method. (b) Primer design and locations of ASLAMP method. (c) Starting structure for self-extension of LAMP. (d) Starting structure for self-extension of ASLAMP.

**Supplementary Figure S2** B-ASLAMP assays of *Shigella* gene were carried out in the presence of 10, 10^2^, 10^3^, 10^4^, 10^5^ and 10^6^ copies of genomic samples. NC is the abbreviation of negative control.
